# A Review of Natural and Synthetic Chalcones as Anticancer Agents Targeting Topoisomerase Enzymes

**DOI:** 10.3390/molecules30122498

**Published:** 2025-06-06

**Authors:** François-Xavier Toublet, Aurélie Laurent, Christelle Pouget

**Affiliations:** Univ. Limoges, LABC*i*S, UR 22722, Faculty of Pharmacy, F-87000 Limoges, France; francois-xavier.toublet@unilim.fr (F.-X.T.); aurelie.laurent@unilim.fr (A.L.)

**Keywords:** natural chalcones, synthetic chalcones, chalcone hybrids, topoisomerases, cancer

## Abstract

Cancer remains one of the leading causes of morbidity and mortality worldwide, driving the search for innovative and selective therapeutic agents. Topoisomerases I and II are essential enzymes involved in key cellular processes such as DNA replication and transcription. They have emerged as valuable anticancer targets; thus, many inhibitors of topoisomerases have been designed and some of them are considered to be major anticancer agents such as anthracyclines, etoposide or irinotecan. A great deal of attention is currently being paid to chalcones, a class of naturally occurring compounds, since they exhibit a wide range of biological activities, including anticancer properties. These compounds are characterized by an open-chain structure and an α,β-unsaturated carbonyl moiety that enables interaction with cellular targets. Recent studies aiming to design anti-topoisomerase agents have identified both natural and synthetic chalcones, including chalcone-based hybrids. This review highlights the structural diversity of chalcones as topoisomerase inhibitors and particular attention is given to structure–activity relationship studies and molecular hybridization strategies aimed at optimizing the pharmacological profile of chalcones. These findings underline the potential of chalcones as promising scaffolds in the design of next-generation anticancer agents.

## 1. Introduction

The latest GLOBOCAN estimates, produced by the International Agency for Research on Cancer (IARC) in 2022, announced 20 million new cancer cases and 9.7 million cancer deaths worldwide [1]. Considering these statistics, cancer is expected to be the leading cause of death in the 21st century. Consequently, major goals of current research are the development of more efficient and specific chemotherapeutics as well as advances in targeted therapies with regard to novel biological targets. Among the latter, DNA topoisomerases are of considerable interest since they are involved in the regulation of the three-dimensional structure of DNA, which is important for the handling of DNA during vital cellular processes such as replication, transcription, chromosome segregation and recombination [2,3].

Control of the DNA topological state by topoisomerases is performed through transient DNA cuts using a transesterification reaction, which is highly reversible. These enzymes can be classified into two main types according to their mechanism of action [2].

Type I topoisomerases cleave single DNA strand whereas type II enzymes break both strands. Topoisomerases I can be divided into three subfamilies: topoisomerases IA, IB and IC [2]. For all these subtypes, controlling the DNA topology by the phosphodiester bond breakage between DNA strands is based on the following mechanism: the phosphoryl group of DNA is attacked by the tyrosyl group of topoisomerase I (topo I), creating a covalent bond between this tyrosyl group and one side of the broken DNA. This allows the free hydroxylated strand to rotate. Then, the hydroxyl termination of the free strand of DNA attacks the phosphotyrosine bond and remakes the phosphodiester bond between the two strands, releasing the topoisomerase to start the next catalytic cycle. Topo I inhibitors mechanism of action implies the entrapment of a newly created topo I–DNA covalent complex and prevents its rupture. This leads to the permanent disruption of the DNA strands and therefore, to cell cycle arrest and apoptosis induction. Camptothecin (CPT, **1**, Table 1) is the first identified example of a topo I inhibitor (IC_50_ = 5.7 µM) [4,5]; it is a natural compound with a pentacyclic structure isolated from the bark of the *Camptotheca acuminata* tree. Two semi-synthetic derivatives have been developed and serve as anticancer drugs in chemotherapy protocols: these are topotecan (**2**, IC_50_ = 3.2 µM) and irinotecan [4,6].

Type II topoisomerases, meanwhile, are divided into two subfamilies: topoisomerases IIA and IIB [2]. The general mechanism of DNA topology control by type II topoisomerases is based on cleaving both strands of the DNA duplex. Topo II covalently binds tyrosine to the 5′ end of broken DNA, releases a free 3′ end and allows it to pass a second DNA duplex through the gap. Currently, two types of topo II inhibitors have been described: topoisomerase poisons and catalytic inhibitors. Topo II poisons act through inhibition of topo II’s ability to religate the cleaved DNA strands. This mechanism of action is based on blocking the rejoining of the broken DNA ends as a result of slipping the topo II poison agents between the separated nitrogenous bases. Well-known examples of topo II poisons are the anthracyclines, including doxorubicin (DOX, **3**, IC_50_ = 0.9 µM) and epirubicin [4,7]. Despite their great anticancer properties, anthracyclines are responsible for a serious side effect, dose-dependent cardiotoxicity. Another important topo II poison is etoposide (**4**), which is a glycosidic podophyllotoxin derivative (IC_50_ = 34.5 µM) [4,5]. Catalytic inhibitors are the second type of topo II inhibitors but there are few examples of these inhibitors in the literature. Dexrazoxane and merbarone may be cited [2]. These agents are a heterogeneous group of compounds that might interfere with the binding between DNA and topo II, stabilize noncovalent DNA–topo II complexes, or inhibit ATP binding.

Chalcones, of the family of flavonoids, constitute a major group of natural compounds that display a wide range of biological activities, including anti-inflammatory [8], antimicrobial [9,10], antioxidant [11] and anticancer properties [12]. Moreover, chalcones are the immediate precursors in the biosynthesis and synthetic preparation of other classes of flavonoids, which are also known for their numerous beneficial effects [13].

**Figure 1:**
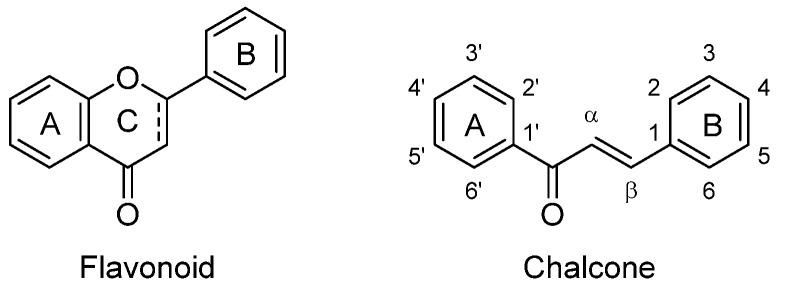
General structures of flavonoids and chalcones.

Unlike flavonoids, chalcones are open-chain molecules with a structure based on a 1,3-diarylprop-2-en-1-one skeleton (Figure 1). The three-carbon enone fragment, which joins the two aromatic rings, can be considered as a Michael acceptor [14]. This explains the ability of chalcones to interact with many biological targets. Indeed, the α,β-unsaturated carbonyl functional moiety participates in covalent bond formation with thiols through Michael addition, especially with cysteine units present in certain proteins or enzymes. Besides, it is also worth considering the substituents of aromatic rings, which may have a double influence:

- toward the electron density on the ring and consequently the electrophilicity of the α,β-unsaturated ketone system, affecting the binding ability and the biological activity of chalcones;
- on the capacity of chalcones to establish hydrogen bonds with pertinent residues (amino acids) of proteins or active sites of enzymes.

Thus, for many years, chalcones have been demonstrated to be efficient topoisomerases inhibitors even if there is still no clinically available antitumor drug with a chalcone structure. In this review, we focused on the biological effects of chalcone derivatives as topoisomerase inhibitors reported in recent years and we have classified them according to two families: natural chalcones and synthetic chalcones, including chalcone-based hybrids.

**Table 1:** Known natural topoisomerase inhibitors.

C. No (Name)	Structure	Activity on Topo: IC_50_ (µM) or % Inhibition	Activity on Cancer Cells IC_50_ (µM)	Ref.
**1** (camptothecin)	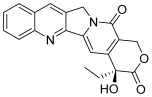	Topo I: 5.7 µM 72% at 100 µM 31% at 20 µM	HCT15: 7.1 BT474: 7.0 T47D: 4.1	[5,15]
**2** (topotecan)	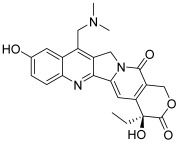	Topo I: 3.2 µM Topo IIB: 99.1 µM	HCT116: 12.2 SR: 13.4	[6,16]
**3** (doxorubicin)	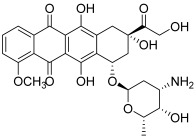	Topo II: 0.9 µM	Caco-2: 0.7 Hep-2: 0.5 HepG2: 0.6	[7]
**4** (etoposide)	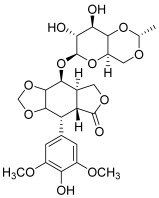	Topo II: 34.5 µM 78% at 100 µM 39% at 20 µM	HCT15: 6.9 BT474: 6.6 T47D: 6.4	[5,15]

## 2. Natural Chalcones as Topoisomerase Inhibitors

Chalcones are widely distributed in the plant kingdom and generally have hydroxyl or methoxy substituents on their aromatic rings. However, other types of substitution are also commonly observed, such as prenyl and geranyl moieties. Here, we report natural chalcones as topoisomerases inhibitors considering two classes, monomeric chalcones and oligomeric ones (Table 2).

### 2.1. Natural Monomeric Chalcones

Yoon et al. revealed an inhibitory effect of licochalcones A and E (**5** and **6**) toward topo I (dose-dependent inhibition in the range of 18.5 to 295.5 µM), in addition to an antiproliferative activity against human cancer cell lines (A549: lung, SK-OV-3: ovary, SK-MEL-2: melanoma and HCT-15: colon) [17]; however, the exact cytotoxic and anti-topo mechanisms remain to be investigated. Licochalcones A and E correspond to retrochalcones, i.e., chalcones lacking hydroxy groups at the 2′ and 6′ positions. These compounds are present in the *Glycyrrhiza* genus, for example in *Glycyrrhiza inflata*.

The *Glycyrrhiza* genus includes also *Glycyrrhiza glabra* (named licorice) and *Glycyrrhiza uralensis*, which are found in common foods and Chinese herbal medicines. Isoliquiritigenin (2′,4,4′-trihydroxychalcone, ISL, **7**), which is isolated from licorice root, seems to be one of the most interesting bioactive compounds with a chalcone structure. ISL has been shown to significantly inhibit the viability of numerous cancer cells, with little toxicity on normal cells [18]. Many studies have focused on the targeted pathways and molecular mechanisms of ISL against tumors. Thus, Zhao et al. demonstrated that in glioma U87 cells, ISL was able to induce cell apoptosis, especially through the inhibition of topo I; the inhibitory effect became obvious in the DNA electrophoretogram when the concentration reached up to 2.5 µM [19]. Besides, it was found that ISL also blocks DNA cleavage reactions by inhibiting topo I activity (IC_50_ = 178 µM, measured by an in vitro enzymatic assay) as well as the growth of SNU475 cells, which are from hepatocellular carcinoma [20]. These two studies mentioned performing molecular docking work; for both studies, it appeared that binding of ISL to topo I was based mainly on hydrophobic interactions. It was shown that ISL bound to the active central pocket of topo I, close to the amino acid residues (Arg364, Asn352, Tyr723 and Thr718) of the catalytic center. Moreover, one hydroxyl group on the A ring formed a hydrogen bond with residue Tyr723. This inhibitory effect of ISL was confirmed by the study of Zhang et al., which concerned secondary metabolites from *Isodon ternifolius* and their anticancer activities [21]. ISL displayed a moderate anti-topo I activity as did a retrochalcone named echinatin (**8**, IC_50_ = 45 µM); moreover, these two chalcones showed a synergistic effect with topotecan against the proliferation of breast cancer MCF-7 cells.

Another important natural chalcone is millepachine (**9**), which was initially extracted from the seeds of *Millettia pachycarpa* Benth.; the presence of a 2,2-dimethylbenzopyran pattern gives it structural originality and increased lipophilicity for efficient membrane penetration [22]. This natural compound was found to exert a wide range of anticancer activities [23]. Among the latter, topo II appeared as a biological target of millepachine. In 2016, Wu et al. first demonstrated that this chalcone was able to induce apoptosis in two ovarian cancer cell lines (SK-OV-3 and A2780S cells). Furthermore, they proved, through the use of a topo II drug screening kit, its inhibitory effect against topo II (IC_50_ < 100 µM). A computer modeling study allowed them to investigate how millepachine interfered with topo II; in fact, this chalcone acted by binding to the topo II-cleaved DNA complex to stabilize it; so, millepachine could be considered as a topo II poison [24]. It was found that there were some π–π interactions between aromatic rings of millepachine and DG-12/DG-13 nucleotides (DG: deoxyguanosine); moreover, the oxygen atom of 2*H*-benzopyran formed a hydrogen bond with the amine of DC-8 nucleotide (DC: deoxycytosine). The same authors continued their investigation, showing that millepachine significantly inhibits the proliferation of cisplatin-resistant A2780CP cells (human ovarian cancer), still via inducing apoptosis and down-regulating the activity of topo II (dose-dependent inhibition in the range 2 to 8 µM) [25].

Xanthohumol (**10**) is a prenylated chalcone isolated from hops that demonstrated a wide range of biological activities [26]. Among them, the anticancer effect of xanthohumol was reviewed in 2021 by Harish et al., and this chalcone showed promising bioactivities on different cancers [27]. Moreover, several signaling pathways were found to be affected; thus, Lee et al. revealed a clear inhibition of topo I activity with a concentration of 140 µM, campthotecin being the positive control [28]. Besides, xanthohumol was shown to exert a strong cytotoxic effect against A549, HCT-15, SK-OV-3 and SK-MEL-2.

**Figure 2:**
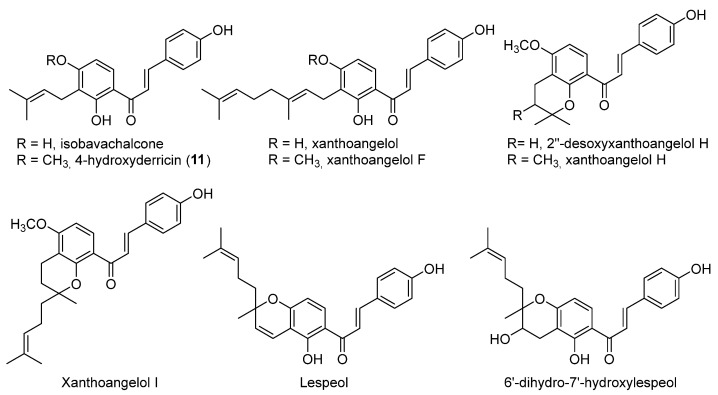
Structures of chalcones isolated from *Angelica keiskei* root extract. Adapted from Ref. [5].

Another prenylated chalcone, named 4-hydroxyderricin (**11**), was found to inhibit topo II (IC_50_ = 21.9 µM) but not topo I activity [5]. This compound was isolated from *Angelica keiskei* roots, with eight other chalcones (Figure 2). 4-Hydroxyderricin was the only one to exert an anti-topo effect and was the most potent cytotoxic agent against four human cancer cell lines (HL-60, CRL1579 melanoma, A549 and AZ521 stomach). This chalcone was found to induce apoptotic cell death in HL60 cells via both the death receptor-mediated pathway and the mitochondrial one. In comparison to this compound, isobavachalcone with a 4′-hydroxy instead of a 4′-methoxy, was totally inactive against topoisomerases as were the other chalcones. The latter were characterized by a geranyl moiety (xanthoangelol and xanthoangelol F) or by a 3,4-dihydrobenzopyran pattern (derivatives of xanthoangelol H and I, and lespeol). This heterocycle came from the cyclisation of the prenyl or geranyl moiety, as found in the millepachine structure. It is worth noticing some structural differences, which seem to influence the activity against topoisomerases:

- xanthoangelol F bearing a geranyl moiety was inactive while 4-hydroxyderricine, with a prenyl substituent, was able to significantly inhibit topo II;
- derivatives of xanthoangelol H, which were inactive against topoisomerases, are characterized by a 3,4-dihydrobenzopyran ring and a 4-hydroxy group, compared to millepachine, a topo II inhibitor, which presents a benzopyran and a 4-methoxy substituent.

In 2023, Savaspun et al. isolated flavokawain B (**12**) from *Kaempferia elegans* rhizomes [29]. This chalcone was found to have cytotoxic activity against hepatocellular carcinoma (HepG2), acute lymphoblastic leukemia (MOLT-3), cholangiocarcinoma (HuCCA-1), and lung carcinoma (A549) cancer cells (IC_50_ between 10.0 and 21.7 µM). Molecular modeling tests demonstrated that flavokawain B was able to present interactions with topo IIA, similar to those of etoposide. More precisely, the docking experiment exhibited π–π interactions with DNA residues, DG-13 and DC-8, as etoposide does and as previously described for millepachine. The strong π–π interactions could be owing to the electron-donating groups, i.e., methoxy groups that increase the electron density of the aromatic ring. Further biological tests are required to validate these data.

Most natural chalcones seem to present a moderate inhibitory effect against topoisomerases while their cytotoxic activity is often significant toward many cancer cell lines. Therefore, different mechanisms of action may be involved in addition to the anti-topo effect to explain these outcomes. 4-hydroxyderricin appeared to be one of the most potent natural chalcones, with strong activity against topo II. Its structure is characterized by hydroxy and methoxy groups as well as a prenyl substituent. These data suggest that lipophilicity is critical for efficient membrane penetration in order to have an anti-topo effect. Besides, oxygenated groups appear essential, particularly for establishing hydrogen bonds with amino acid residues of the topo active site or π–π interactions with DNA residues. The comparison between 4-hydroxyderricin and isobavachalcone demonstrates that simply replacing a methoxy with a hydroxy can reverse the inhibitory effect against topoisomerases. This is also the case for replacement of the prenyl substituent by a geranyl one. So, we may suggest that too much lipophilicity is unfavorable for the activity. This study also revealed the potency of millepachine, another methoxylated chalcone bearing a 2,2-dimethylbenzopyran pattern involved in lipophilicity as well as hydrogen bond formation through the oxygen atom with DNA nucleotides. Results of computer modeling demonstrate that this chalcone could bind to the topo II-cleaved DNA complex to stabilize it, indicating a topo II poison effect.

### 2.2. Natural Oligomeric Chalcones

Some original chalcones, having oligomeric forms, were found to act as topoisomerase inhibitors. Pauferrol A (**13**) is an example of a chalcone trimer fused by a cyclobutane ring and was isolated from *Caesalpinia ferrea*, a leguminous plant in Brazil. This compound has been reported to possess a topo II inhibitory effect (IC_50_ = 2.1 µM) and apoptosis inducing activity in the human acute myeloid leukemia HL-60 cell line [30]. Later, the same plant revealed the presence of two new chalcone dimers, pauferrol B (**14**) and pauferrol C (**15**). They also showed inhibitory potencies against human topo II (IC_50_ = 15.3 and 14.5 µM) and cell proliferation via the induction of apoptosis in HL-60 cells but they were significantly less active than pauferrol A [31]. Then, Aljancic et al. isolated a chalcone dimer, fused by a cyclobutane ring, from *Helichrysum zivojinii* [32]. This compound, named tomoroside A (**16**), was studied considering its effect on the level of topo IIA mRNA expression in NCI-H460 and NCI-H460/R (resistant to paclitaxel and DOX drugs) lung cancer cells. This expression was really decreased after treatment with tomoroside A; moreover, the chalcone dimer enhanced the DOX anticancer effect.

Finally, Yue et al. investigated the biological properties of quinochalcones isolated from *Carthamus tinctorius* L. by Kazuma et al. [33,34,35]. These compounds include anhydrosafflor yellow B (also known as carthorquinoside B, **17**), which consists of two C-glucosylquinochalcone moieties with an additional sugar part. It showed a moderate inhibitory effect on topo I (inhibition at 100 µM, no lower dose tests). Furthermore, it revealed an antiproliferative effect on many cancer cell lines with an inhibition rate of over 50% at 9.6 μM. Therefore, this highly original molecule was patented by the Nanjing University of Chinese Medicine [36].

As a partial conclusion, some oligomeric chalcones, particularly pauferrols that are polyhydroxylated compounds, seem to be interesting as topoisomerase inhibitors since they reveal IC_50_ values lower than that of monomeric chalcones like ISL (2′,4,4′-trihydroxychalcone).

**Table 2:** Natural chalcones as topoisomerases inhibitors.

C. No (Name)	Structure	Activity on Topo: IC_50_ (µM) or % Inhibition	Activity on Cancer Cells IC_50_ (µM)	Ref.
**5** (licochalcone A)	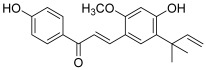	Topo I: dose-dependent inhibition 18.5 to 295.5 µM	A549: 14.3 HCT-15: 10.1 SK-OV-3: 13.5 SK-MEL-2: 7.9	[17]
**6** (licochalcone E)	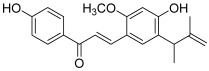		A549: 17.3 HCT-15: 10.1 SK-OV-3: 15.5 SK-MEL-2: 8.5	[17]
**7** (isoliquiritigenin)	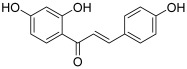	Topo I: 178 µM	SNU475: 243 U87: 6.3	[19,20]
**8** (echinatin)	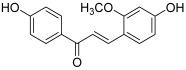	Topo I: 45 µM	A549: 36.0 HCT116: 64.0 MCF-7: 46.0	[21]
**9** (millepachine)	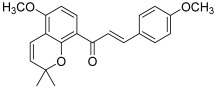	Topo II: <100 µM dose-dependent inhibition 2 to 8 µM	A2780CP: 4.0 A2780S: 2.5 SK-OV-3: 4.0	[24,25]
**10** (xanthohumol)	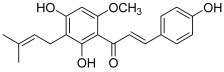	Topo I: inhibition of activity with a concentration of 140 µM	A549: 12.1 SK-MEL-2: 14.4 HCT115: 10.2 SK-OV-3: 16.1	[28]
**11** (4-hydroxyderricin)	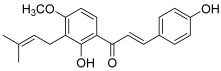	Topo I: No activity Topo II: 21.9 µM	A549: 10.2 AZ521: 4.2 CRL1579: 4.6 HL-60: 5.5	[5]
**12** (flavokawain B)	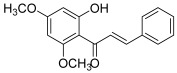	Topo IIA	A549: 21.7 HepG2: 20.7 HuCCA-1: 19.6 MOLT-3: 10.0	[29]
**13** (pauferrol A)	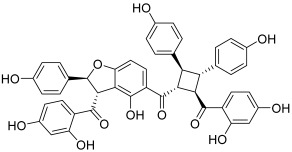	Topo IIA: 2.1 µM	HL-60: 5.2	[30]
**14**–**15** (pauferrol B and C)	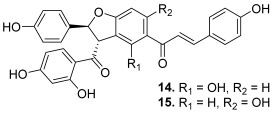	Topo IIA: 14. 15.3 µM 15. 14.5 µM	HL-60: 14. 11.6 15. 12.1	[31]
**16** (tomoroside A)	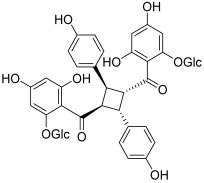	Topo IIA: decreased mRNA expression in cancer cells	NCI-H460: 44.4 NCI-H460/R: 199	[32]
**17** (carthorquinoside B)	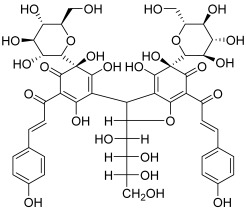	Topo I: inhibition at 100 µM (no lower dose tests)	HeLa, HepG-2, A549, K562, and HCT116: between 3.13 and 200	[34]

## 3. Synthetic Chalcones as Topoisomerase Inhibitors

In this section, we will first present the studies concerning synthetic chalcones including variously substituted chalcones, heterocyclic chalcones and chalcone–metal complexes. Then, investigations dealing with chalcone hybrids as topoisomerase inhibitors will be reported.

### 3.1. Synthetic Chalcones

**Scheme 1:**
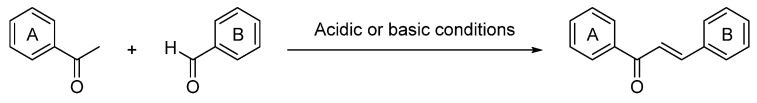
Synthesis of chalcones by Claisen–Schmidt condensation.

Natural chalcones often present hydroxyl or methoxy substituents on their aromatic rings; therefore, several works were carried out around the modification of methoxylated or hydroxylated chalcones. Some other studies have focused on the introduction of heterocycles or their complexation with different metals. All of these chalcones were synthesized by Claisen–Schmidt condensation, most often in basic conditions, between appropriate acetophenones and benzaldehydes (Scheme 1). Structures and biological evaluation of these synthetic chalcones are reported in Table 3.

In 2019, Sangpheak et al. performed docking studies and tested 47 chalcones on the ATP-binding pocket of the hTopo IIA ATPase domain [37]. There are two important patterns for drugs targeting hTopoIIA, namely the ATPase domain and the DNA-binding core. The resulting data highlighted three polymethoxylated chalcones that were synthesized and biologically tested. All these derivatives had a 2-hydroxy-4,6-dimethoxy substitution pattern on A ring while their B ring was trimethoxylated at different positions. First, the cell viabilities of three cancer cell lines: HT-1376 (human bladder carcinoma), HeLa (cervical cancer metastasis) and MCF-7, exposed to the selected chalcones, were determined by an MTT assay; the most cytotoxic was the chalcone bearing a 3,4,5-trimethoxyphenyl as its B ring (**18**). So, its inhibitory activity was evaluated by measuring the ATPase activity of rhTopo IIA ATPase, with salvicine (**19**, catalytic inhibitor) as the reference compound [38]. The IC_50_ value of this chalcone was some 43.5-fold lower than that of salvicine (IC_50_ = 7.5 and 326.5 nM). Some important residues that contributed to ligand stabilization via van der Waals and H-bond interactions were identified, like K168, which is in common with salvicine, and residues S149 and G164, which form two strong H-bond interactions with the carbonyl and 3-methoxy groups of chalcone **18**.

Recently, Chen et al. screened more than three hundred synthetic, semisynthetic or naturally occurring chalcones for inhibition of the TOP2A/Wnt/β-catenin signaling [39]. The Wnt/β-catenin pathway implies a family of proteins that play critical roles in embryonic development and adult tissue homeostasis. Persistent activation of this pathway is common in carcinogenesis, such as colorectal cancer (CRC) and hepatocellular carcinoma. Moreover, it was demonstrated that topo IIA acts as a co-activator of β-catenin, thus increasing the transcription of oncogenes. Among the whole chalcones tested, one derivative (5′-chloro-3,4-ethylenedioxy-2′-methoxychalcone, **20**) was selected because it inhibited the in vitro cell growth of well-known CRC cell lines. Further assays highlighted this chalcone to be a novel inhibitor of TOP2A/Wnt/β-catenin signaling (decreased mRNA expression at 5 µM). Therefore, it has been patented by the University of Kentucky Research Foundation, where the researchers came from [40].

Another work has focused on the modification of hydroxylated chalcones, particularly in the 4′-position. In 2009, Gul et al. synthesized a small series of 4′-hydroxychalcone derivatives to study their cytotoxic activity against transformed human T lymphocytes (Jurkat cell line) as well as their effect on mammalian topo I [41]. The B ring of these compounds was *para*-substituted with a methyl, a methoxy or a chlorine atom. Their activities were compared to those of 4′-hydroxychalcone, the parent chalcone. An additional chlorine atom appeared to be favorable since 4-chloro-4′-hydroxychalcone (**21**) was the most efficient against both cellular proliferation and topo I activity (76% inhibition at 3.9 mM). Nevertheless, this inhibitory effect could be described as weak compared to that of other compounds.

Kim et al. also investigated the effect of different chalcones, especially 4′-hydroxychalcones, against topoisomerases I and II [42]. A Claisen–Schmidt reaction performed without protection of the 4-hydroxy group on acetophenone and/or benzaldehyde gave unsatisfactory yields; so, a protection step was carried out using a tetrahydropyranyl group, allowing a significant increase of the obtained yields. First, unlike a chlorine atom (in compound **21**), an additional fluorine atom was found to decrease the inhibitory effect toward topo I (topo I: 51% and 18% inhibition at 100 and 20 µM; topo II: 0% inhibition at 100 µM). Secondly, within the series, other compounds were more interesting against topo I whereas most were inactive on topo II. The derivative bearing a 1-pyrrolidine (**22**) as the substituent on the 4-position of the B ring was the most potent since it was more active than camptothecin at 20 µM against topo I, while also being a weak inhibitor of topo II (topo I: 75% and 60% inhibition at 100 and 20 µM respectively; topo II: 19% inhibition at 20 µM). Moreover, this chalcone was found to be the most cytotoxic agent against T47D (human breast ductal carcinoma) and SNU638 (human gastric cancer) cells. The whole compounds synthesized have been patented by Ewha University [43].

Then, Na and Nam designed some original chalcones through the introduction of epoxide or thioepoxide moieties on a hydroxylated chalcone scaffold [44]. Their study was based on their previous results, which showed that substitution of xanthones with epoxide or thioepoxide fragments could generate inhibitory activity on topoisomerase I or II [45,46]. The same strategy as previously described allowed them to obtain some hydroxy-chalcones, which were treated with excess amounts of epichlorohydrin or epithiochlorohydrin under basic conditions. The most potent compound synthesized by Na and Nam was a chalcone with a bis-thioepoxypropoxy substitution pattern on the A ring and a 4-methoxyphenyl as the B ring (**23**). This compound strongly inhibited MCF-7 and HCT-15 cancer cells proliferation (IC_50_ 0.49 and 0.23 µM, respectively). Moreover, it inhibited topo II activity with an extent comparable to etoposide at a 100 µM concentration (topo I: 61% and 21% inhibition at 100 and 20 µM, respectively; topo II: 0% inhibition at 100 µM).

In 2016, the previous team published additional results, keeping a thioepoxypropoxy or epoxypropoxy on the 2′, 3′ or 4′ position of the A ring, but changing the B ring in the form of a thiophene or furan. In this article, Jeon et al. synthesized 22 chalcones using the same synthetic pathway as described by Na and Nam [47]; four of these molecules (**24**–**27**) were found to be topoisomerase inhibitors. Compounds **24**, **25** and **26** (A ring with a thioepoxypropoxy and B ring: 2-furan) presented selective activity against topo II (around 90% and 30% inhibition at 100 and 20 µM, respectively) and were superior to etoposide (72% and 18% inhibition at the same concentrations). Compound **24** inhibited T47D cancer cells growth while compound **26** suppressed MDA-MB-468 (breast) cancer cells proliferation. As for the chalcone **27**, with a 2′-epoxypropoxy-substituted A ring and a furan-2-yl as the B ring, it presented the highest activity against topo I, but lower than CPT. So, replacement of an epoxide by a thioepoxide was likely to modify the selectivity for topo I or topo II; thus, at 20 µM, percentages of inhibition for compound **24** were 6% and 31% against topo I and topo II, respectively, while for chalcone **27**, they were equal to 24% and 0%, respectively. Moreover, having a thiophene or a furan-3-yl instead of a furan-2-yl completely suppressed the inhibitory effect.

Heterocycles seem to be interesting in terms of modulation of the chalcone structure. Thus, Kasetti et al. designed 20 thiazole-chalcones, considering that this heterocycle was pertinent to favorably influence both the pharmacokinetic and pharmacodynamic properties of the resulting drugs [48]. Synthesis was performed through the condensing of different aromatic ketones with 2,4-dichlorothiazole-5-carboxaldehyde, but in acidic conditions. This series was tested on a prostate cancer cell line (DU145). Only the molecule **28,** for which both A and B cores corresponded to a thiazole moiety, had an IC_50_ of less than 10 µM, i.e., inferior to that of methotrexate (the reference molecule in this article). The series was further investigated through molecular modeling studies on topo IIA ATPase, chalcone **28** obtaining the highest score in terms of binding affinity. H-bond interactions with the Asn95, Ser149, Asn150, and Arg168 and hydrophobic interactions with the Arg98 and Ala167 amino acid residues were highlighted. Nevertheless, these promising results have to be confirmed by biological tests.

Instead of classic hydroxy substituents, Santos et al. were interested in introducing amino groups on the chalcone core [49]. They synthesized 15 chalcones featuring a NH_2_ group in the 2′ or 4′ position with various B rings. The compounds were tested on a canine malignant histiocytic cell line (DH82). Within the series, three chalcones (4′-amino/4-fluoro, 4′-amino/4-chloro and 2′-amino/4-methyl) appeared as the most active with IC_50_ values ranging from 31 to 38 µM. Thus, these molecules were more efficient than etoposide and then they were tested on topo IIA. Only the 2′-amino-4-methylchalcone (**29**) showed an inhibitory effect toward transcription of the TOP2A and TP53 genes (decreased mRNA expression in cancer cells at 40 µM).

Then, a study by Štefanišinová et al. described one chalcone and three chalcone-like compounds, with a single substituent: a 4-*N*,*N*-dimethylamino group (**30**) [50]. This study focused on the interaction between chalcones and DNA using spectroscopic techniques and topo I relaxation assays. The whole molecules were demonstrated to inhibit topo I at 60 µM.

Finally, some studies were carried out to evaluate the potency of chalcone–metal complexes. Thus, Gaur and Mishra developed chalcone or bis-chalcone complexes with ruthenium. C*is*-[Ru(*S*-DMSO)_3_(chalcone)Cl] type complexes were synthesized and characterized using spectroscopic and single crystal X-ray diffraction techniques [51]. Ruthenium complexes are well-known for their antitumor activity, in particular *cis*-Ru(DMSO)_4_Cl_2_, owing to its stabilization using heteroaromatic ligands [52,53]. Two complexes from Gaur and Mishra were proven to strongly interact with DNA. These are complexes of *cis*-[Ru(*S*-DMSO)_3_(chalcone)Cl]: 2′-hydroxyphenyl as the A ring and 2-thiophenyl (**31**) or 3-methyl-2-thiophenyl (**32**) as the B ring. Both Ru(II) complexes inhibited the activity of topo II at a low concentration (IC_50_ = 13–18 μM), comparable to some classical topo II inhibitors. To go further, they looked at Ru(II) complexes of bis-chalcones, developing complex **33**, which showed moderate potential for inhibiting, this time, topo I (IC_50_ = 87 μM) [54].

In addition, some ferrocenyl chalcone-like derivatives were designed and investigated to evaluate their ability to inhibit the activity of topoisomerases I/II [55]. Many reports have demonstrated that ferrocenyl derivatives may be greatly cytotoxic against numerous cancer cell lines, having also a possible topoisomerase inhibitory effect [56]. The study of Konkolova et al. pointed out the potency of three ferrocenyl chalcone-like compounds against topo I; among them, two complexes (**34**–**35**) were found to also inhibit topo II activity (partial inhibition at 30 and 5 µM, respectively) [55].

As a discussion from the previous results concerning these synthetic chalcones, some structure–activity relationship elements can be highlighted. First, the potential of methoxy groups may be confirmed by different studies, like those of Sangpheak et al. or Na and Nam [37,44]. The introduction of heterocycles on the chalcone core also appears as a positive way to obtain inhibitory effects against topoisomerases. Then, molecular modeling studies could be pertinent to explain some results, for example, those of Jeon et al. concerning the nature of heterocycles, which influences either the activity or the selectivity toward topoisomerases. It is worth noticing that a chalcone–ruthenium complex with a 2-thiophenyl as a B ring was able to significantly inhibit topo II [51], whereas this heterocycle cancels the anti-topo effect for chalcones bearing a thioepoxypropoxy or epoxypropoxy on the A ring.

**Table 3:** Synthetic chalcones and reference substances as topoisomerases inhibitors.

C. No (Name)	Structure	Activity on Topo: IC_50_ (µM) or % Inhibition	Activity on Cancer Cells IC_50_ (µM)	Ref.
**18**	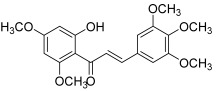	Topo IIA ATPase: 7.5 nM	HeLa: 3.2 HT-1376: 10.8 MCF-7: 21.1	[37]
**19** (salvicine)	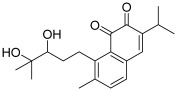	Topo IIA ATPase: 326.5 nM	HeLa: 70.1 HT-1376: 106.5 MCF-7: >200	[37,38]
**20**	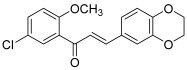	Topo IIA: decreased mRNA expression at 5 µM	DLD-1: 0.3 HCT116: 0.3 HT-29: 0.7 LS174T: 0.4	[39]
**21**	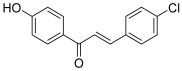	Topo I: 76% at 3.9 mM	Jurkat: 9.3	[41]
**22**	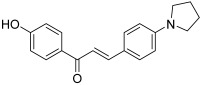	Topo I: 75% at 100 µM 60% at 20 µM Topo II: 97% at 100 µM 19% at 20 µM	SNU638: 0.6 T47D: 1.4	[42]
**23**	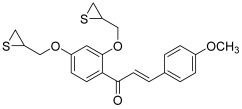	Topo I: 0% at 100 µM Topo II: 61% at 100 µM 21% at 20 µM	DU145: 1.1 HCT15: 0.2 K562: 7.6 MCF-7: 0.5	[44]
**24**	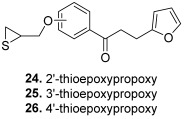	Topo I: 61% at 100 µM 6% at 20 µM Topo II: 95% at 100 µM 31% at 20 µM	MDA-MB-231: 32.2 MDA-MB-468: 8.3 T47D: 6.6	[47]
**25**		Topo I: 75% at 100 µM 1% at 20 µM Topo II: 90% at 100 µM 30% at 20 µM	MDA-MB-231: 28.9 MDA-MB-468: 7.2 T47D: 10.4	
**26**		Topo I: 64% at 100 µM 0% at 20 µM Topo II: 95% at 100 µM 25% at 20 µM	MDA-MB-231: 18.0 MDA-MB-468: 4.3 T47D: 9.4	
**27**	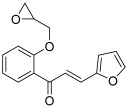	Topo I: 37% at 100 µM 24% at 20 µM Topo II: 82% at 100 µM 0% at 20 µM	MDA-MB-231: 28.7 MDA-MB-468: 48.3 T47D: 19.4	[47]
**28**	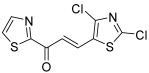	Topo IIA ATPase	DU145: 6.9	[48]
**29**	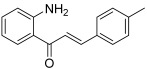	Topo IIA: decreased mRNA expression in cancer cells at 40 µM	DH82: 38.2	[49]
**30**	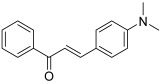	Topo I: inhibition at 60 µM	-	[50]
**31**–**32**	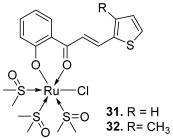	Topo II: 31. 18 µM 32. 13 µM	-	[51]
**33**	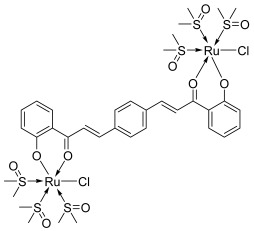	Topo I: 87 µM	-	[54]
**34**–**35**	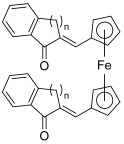	Topo I: partial inhibition at 30 µM Topo II: partial inhibition at 5 µM	-	[55]

### 3.2. Chalcone Hybrids as Topoisomerases Inhibitors

The hybridization of biologically active molecules is a powerful tool for drug discovery. Different objectives are considered through this strategy. Generally, hybridization involves the combination of two (or more) drugs/pharmacophores into a single molecule. Either the goal is to strengthen the potency against one biological target, or it is aimed, more and more often now, to design multi-targeted drugs (MTDs). Indeed, in the field of cancer treatment, the development of MTDs has been under constant escalation for the last two decades. The advantages of MTDs are numerous: they can improve treatment outcomes, lead to synergistic effects, and reduce drug resistance probability and side effects [57]. The design of such MTDs may be based on the framework combination approach, which consists of combining drugs/pharmacophores to develop a new hybrid molecule with activity toward multiple biological targets. The individual partners or molecular components can be assembled covalently to form a molecular matrix by linking, fusing or merging strategies [58]. The structures and biological evaluation of these chalcone hybrids are reported in Table 4.

#### 3.2.1. Fluoroquinolone Hybrids

Fluoroquinolones such as ciprofloxacine (**36**) are major antibacterial agents for which the cellular target is topo II (IC_50_ = 104 µM). Therefore, fluoroquinolones can also be considered as potent anticancer agents [59]. In this part of our review, we report different works dealing with hybrids that combine the ciprofloxacine unit with the chalcone core to design novel topoisomerase inhibitors. This hybridization involves different types of linkers, as described below.

**Scheme 2:**
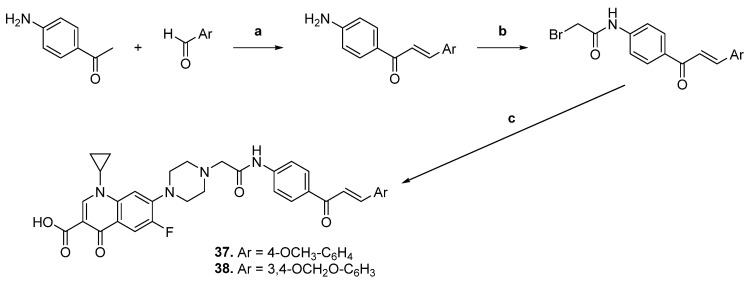
Synthesis of compounds **37** and **38**. Reagents: (**a**) 10% NaOH, EtOH; (**b**) bromoacetyl bromide, K_2_CO_3_, DCM; (**c**) ciprofloxacin, triethylamine, acetonitrile. Adapted from Ref. [15].

In 2013, Abdel-Aziz et al. reported the synthesis of C-7-piperazinyl–ciprofloxacin-chalcone hybrids in order to improve the physicochemical properties of ciprofloxacin and/or to obtain a synergistic effect through combining ciprofloxacin and chalcone in a unique structure [15]. First, chalcone derivatives were synthesized by a base-catalyzed Claisen–Schmidt condensation between 4-aminoacetophenone and different benzaldehydes. Then, treatment of the chalcone intermediates with bromoacetyl bromide in the presence of potassium carbonate produced the corresponding 2-bromo-*N-*{4-[3-arylacryloyl]phenyl}acetamides. Finally, alkylation of ciprofloxacin with the previous acylated chalcones using triethylamine as a base gave the target ciprofloxacin–chalcone hybrids (Scheme 2). So, the ciprofloxacin unit was linked, from the piperazinyl part, to the chalcone moiety through an amide function. These compounds were evaluated for their inhibitory effect against topo I and II enzymes in addition to investigation of their in vitro antiproliferative effect toward several cancer cell lines. Within the series, two compounds (**37**–**38**) revealed similar topo I inhibitory potency at 20 µM compared to CPT used as a positive control (25%, 26% and 31% inhibition, respectively). Considering topo II, the two previous hybrids exhibited a remarkable inhibitory effect compared to etoposide (a positive control for topo II) at both 100 and 20 µM (50%, 44% and 39% inhibition at 20 µM, respectively).

**Scheme 3:**
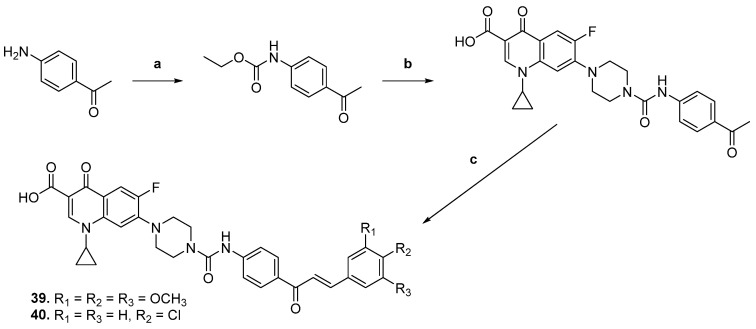
Synthesis of compounds **39** and **40**. Reagents: (**a**) ethyl chloroformate, pyridine, acetonitrile; (**b**) ciprofloxacin, xylene; (**c**) appropriate aromatic aldehydes, 60% NaOH, EtOH. Adapted from Ref. [16].

In 2021, the same research team designed a novel series of urea-linked ciprofloxacine–chalcone hybrids [16]. Once more, the ciprofloxacin unit was linked to the chalcone core from its piperazinyl part. However, the two scaffolds were directly connected to each other through a urea moiety. A carbamate was prepared by stirring 4-aminoacetophenone with ethyl chloroformate using pyridine as a base. Then, ciprofloxacin was refluxed with the previous carbamate intermediate in xylene; condensation of the resulting intermediate with various aromatic aldehydes in ethanolic sodium hydroxide solution led to the target hybrids (Scheme 3). This study revealed the potency of one hybrid compound not only as a dual inhibitor of both topo I and topo II but also as an antiproliferative agent against leukemia and colorectal carcinoma. This compound interestingly possessed a 3,4,5-trimethoxyphenyl (**39**) as the B ring in the chalcone core (IC_50_ = 16 and 137 µM against topo I and II, respectively). This work also pointed out the great interest of another derivative bearing a 4-chlorophenyl (**40**) as the B ring of the chalcone part (IC_50_ = 18.0 and 107 µM against topo I and II, respectively). Thus, this compound was the subject of further investigations in 2024, confirming its inhibitory effects on topoisomerases I and II (IC_50_ = 37.5 and 19.9 µM) and its ability to induce apoptosis in colorectal cancer cells [60].

**Scheme 4:**
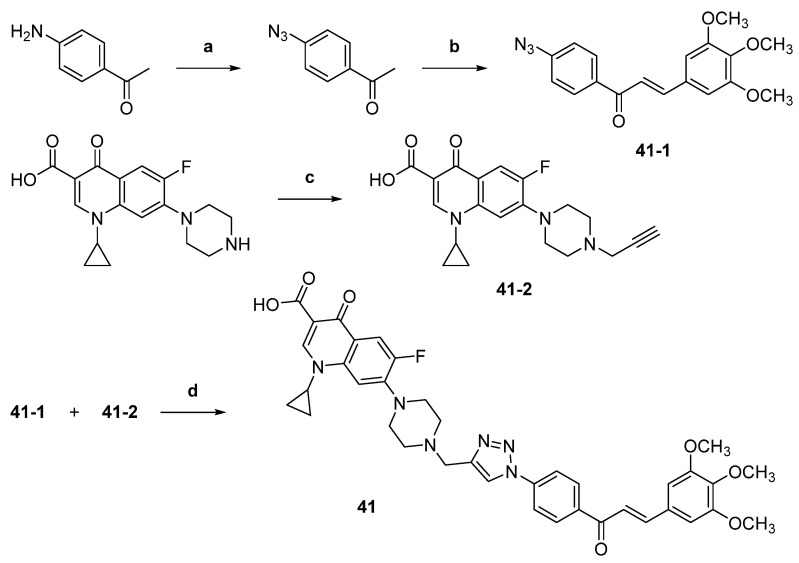
Synthesis of compound **41**. Reagents: (**a**) conc. H_2_SO_4_, NaNO_2_, NaN_3_; (**b**) appropriate aromatic aldehydes, 60% NaOH, EtOH; (**c**) propargyl bromide, NaHCO_3_, DMF; (**d**) CuSO_4_·5H_2_O, sodium ascorbate, DMF, DCM, MeOH, H_2_O. Adapted from Ref. [61].

Finally, the diversification of the linker in this series led these authors to develop new ciprofloxacine–chalcone hybrids, this time with a 1,2,3-triazole linkage [61]. Their synthetic pathway was based on click chemistry using the copper-catalyzed azide-alkyne cycloaddition (CuAAC) between an azido chalcone and a *N-*propargylated (on piperazine heterocycle) ciprofloxacine. More precisely, azido chalcones were synthesized by condensation, in the presence of sodium hydroxide, of the appropriate aromatic aldehydes with 1-(4-azidophenyl)ethanone. The latter was prepared from 4-aminoacetophenone, which was reacted with sodium nitrite in acidic medium at 0 °C; then, an aqueous solution of sodium azide was added. Besides, ciprofloxacin was *N-*alkylated using propargyl bromide in the presence of NaHCO_3_. The final hybrids were synthesized by coupling azido chalcones with the ciprofloxacin derivative using sodium ascorbate and copper sulfate as a catalyst (Scheme 4). As before, the most interesting derivative was still the one with a 3,4,5-trimethoxyphenyl as the B ring in the chalcone core (**41**). In addition to a remarkable anti-proliferative activity against leukemia (RPMI-8226) and colon (HCT116) cancer cells, it exhibited a wide range of inhibitory effects toward topoisomerases I and II (topo I: 25% inhibition at 2.5 µM; topo II: 95%inhibition at 10 µM), but also against tubulin polymerization thanks to the 3,4,5-trimethoxyphenyl moiety (100% inhibition at 2.5 µM) [62].

In brief, these four previous studies demonstrated the potential of ciprofloxacine–chalcone hybrids against both topo I and topo II while ciprofloxacine is only a well-known topo II inhibitor. Different linkers were used to obtain these hybrids and the triazole linker was found to be better than the urea one since compound **41** was more active than hybrid **39**.

**Scheme 5:**
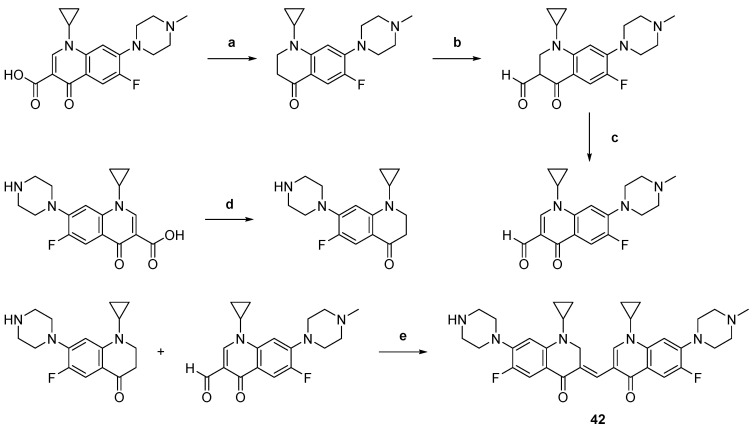
Synthesis of compound **42**. Reagents: (**a**) NaBH_4_, *p*-TsOH; (**b**) ethyl formate, sodium ethoxide; (**c**) MnO_2_; (**d**) NaBH_4_, *p*-TsOH; (**e**) piperidine, EtOH. Adapted from Ref. [64].

To conclude this section on fluoroquinolone derivatives, a final work from Ma et al. is discussed; it concerns a novel bis-fluoroquinolone chalcone-like derivative (**42**) that was synthesized by the Institute of Chemistry and Biology of Henan University (Scheme 5) [63,64]. This latter was designed to retain the structural features of both sunitinib, an inhibitor of tyrosine kinases with an α,β-unsaturated ketone, and fluoroquinolones as topoisomerase inhibitors. This is why this compound has to be considered as a chalcone-like derivative. This one was the first reported dual topo II and tyrosine kinase inhibitor (topo II: partial inhibition at 1.6 µM and virtually complete inhibition at 3.2 μM); moreover, it demonstrated interesting antiproliferative activity against several cancer cell lines, especially pancreatic cells. Considering the effects of this hybrid on topo II activity, the results suggested an increase of topo II-mediated DNA double-strand breaks and inhibition of DNA religation. Finally, Ma et al.’s study reported an example of a merged hybrid with this chalcone-like compound, while all previous works dealt with hybrids containing a linker [15,16,60,61].

#### 3.2.2. Chalcone Hybrids Including Natural Compounds

To continue this review of chalcone hybrids based on a linking strategy, we report studies about hybridization with known natural topoisomerases inhibitors.

**Scheme 6:**
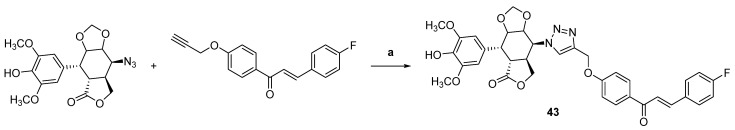
Synthesis of compound **43**. Reagents: (**a**) CuSO_4_, sodium ascorbate, *t*-BuOH, H_2_O. Adapted from Ref. [65].

First, a series of novel epipodophyllotoxin–chalcone hybrids was synthesized by Banday et al. in 2015 [65]. These hybrids consist of structurally different but functionally similar topo II inhibitors. They were conjugated together through a 1,2,3-triazole linker, using click-chemistry by CuAAC between C4-β-azido podophyllotoxins and propargylated chalcones (**43**, Scheme 6).

Evaluation of the antiproliferative activity of these conjugates against a panel of six human cancer cell lines revealed their potency, especially toward SW-620 (colon) and SKN-SH (CNS) cell lines. Then, the compounds were docked against topo II and the glide scores were in good agreement with the observed antiproliferative effect. Moreover, the docking calculations evidenced that the synthesized hybrids have much better binding affinities than etoposide toward topo II.

**Scheme 7:**
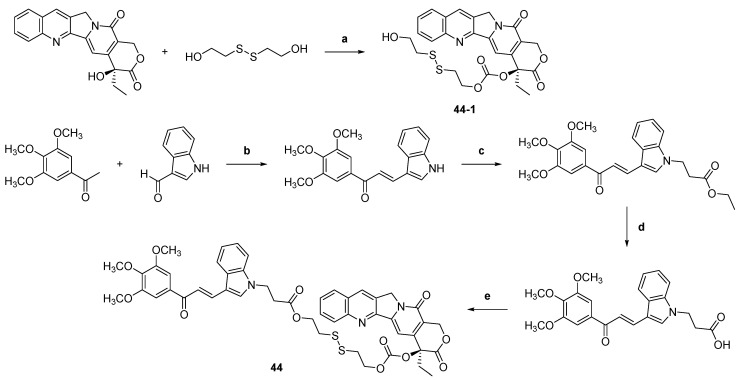
Synthesis of compound **44**. Reagents: (**a**) triphosgene, DMAP, DCM; (**b**) piperidine, EtOH; (**c**) ethyl 3-bromopropionate, K_2_CO_3_, KI, DMF; (**d**) NaOH, MeOH, H_2_O; (**e**) **44-1**, EDCI, HOBt, DMF. Adapted from Ref. [66].

Secondly, Wang et al. reported a series of dual-targeted anti-cancer agents based on indolic chalcone derivatives and the CPT scaffold [66]. The synthetic pathway involved several steps that are described below. First, camptothecin reacted with 2-hydroxyethyl disulfide in the presence of triphosgene and DMAP to afford a carbonate ester of CPT. Then, aldol condensation between 3,4,5-trimethoxyacetophenone and indole-3-carbaldehyde in the presence of piperidine gave indolic chalcones, which reacted with different bromo esters through a nucleophilic substitution. Basic hydrolysis of the intermediate esters led to the corresponding carboxylic acids, which reacted with the carbonate ester of CPT using EDC and HOBT as condensation agents to form hybrid esters between CPT with a disulfide moiety and indolic chalcones (Scheme 7). The latter are known to be potent microtubule destabilizing agents (MDAs) [67] while CPT acts as a topo I inhibitor. In fact, these two parts were attached through disulfide bonds, i.e., a GSH-responsive linker; so, these hybrids must be considered as prodrugs since glutathione (GSH) can reduce the disulfide bonds, resulting in their breakage. Moreover, a correlation between elevated intracellular GSH levels and multidrug resistance (MDR) is well established. Therefore, the design of such compounds is a global strategy to address MDR associated with microtubule destabilizing agents. The different investigations allowed us to demonstrate a considerable antiproliferative activity of one conjugate (**44**) against the tested cancer cell lines, notably paclitaxel-resistant A549 and HCT116 cells. This conjugate was found to operate as a GSH-responsive prodrug, releasing the chalcone moiety and CPT and leading consequently to an inhibition of tubulin polymerization and topo I activity.

#### 3.2.3. Nitrogen Heterocycle-Based Hybrids

**Scheme 8:**
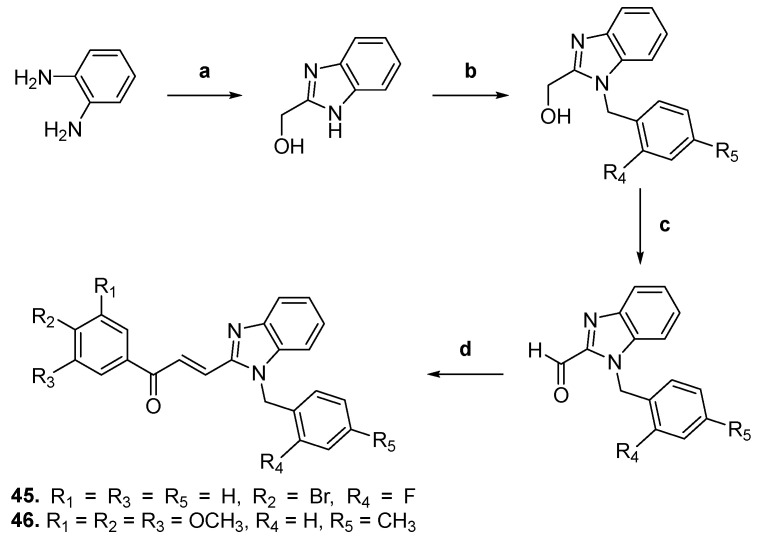
Synthesis of compounds **45** and **46**. Reagents: (**a**) glycolic acid, 4 N HCl; (**b**) appropriate benzyl bromides, K_2_CO_3_, DMF; (**c**) Dess–Martin periodinane, DCM; (**d**) appropriate acetophenones, 10% NaOH, EtOH. Adapted from Ref. [68].

Other chalcone hybrids were designed combining nitrogen heterocycles with a chalcone scaffold. Thus, a series of benzimidazole-chalcone hybrids was synthesized by Zhou et al. [68] since several benzimidazole derivatives have been reported as new topo II inhibitors [69]. These hybrids were obtained in four steps: first, (1*H*-benzo[*d*]imidazol-2-yl) methanol was synthesized by refluxing *o*-phenylenediamine with glycolic acid in hydrochloric acid; this intermediate reacted with appropriate benzyl bromides in the presence of K_2_CO_3_ to give substituted (1-benzyl-1*H*-benzo[*d*]imidazol-2-yl)methanols. Then, these compounds were oxidized using the Dess–Martin reagent in the corresponding benzo[*d*]imidazole-2-carbaldehydes. The target compounds were finally prepared from the previous carbaldehydes and the appropriate acetophenones through the Claisen–Schmidt condensation (Scheme 8). The benzimidazole–chalcone conjugates demonstrated a good inhibitory effect in topo II-mediated DNA relaxation assays and antiproliferative activity against four tumor cell lines: HepG2, A549, LNCaP (prostate) and MG-63 (bone). Within the series, two compounds were identified as the most potent, having IC_50_ values less than 5 µM, superior to etoposide. The first hybrid was characterized by a *N*-2-fluorobenzyl and a 4′-bromophenyl as a chalcone A ring (**45**). The second one was built with a 3′,4′,5′-trimethoxyphenyl as a chalcone A ring and with a *N*-4-methylbenzyl (**46**). Mechanistic studies revealed that these derivatives acted as non-intercalative topo II catalytic inhibitors (87% inhibition for **45**, and 95% inhibition for **46** at 20 µM).

**Scheme 9:**
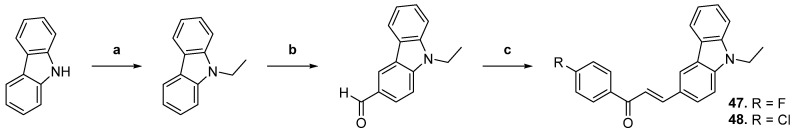
Synthesis of compounds **47** and **48**. Reagents: (**a**) bromoethane, KOH, acetone; (**b**) POCl_3_, DMF, DCM; (**c**) appropriate acetophenones, 10% NaOH, EtOH. Adapted from Ref. [71].

The carbazole scaffold is also of great interest since some studies showed that carbazole derivatives may exert their anticancer effect by topo inhibition [70]. Therefore, the previous researchers designed a series of carbazole derivatives containing chalcone analogs and tested their topo II inhibitory and cytotoxic activities against four cell lines (HeLa, A549, PC-3, and HL-60) [71]. The synthetic pathway involved three steps, the first one being *N*-alkylation of carbazole with appropriate benzyl bromides. The Vilsmeier–Haack reaction from the obtained intermediates led to *N*-substituted 9*H*-3-carbaldehydes, which gave the desired hybrids after aldol condensation with appropriate acetophenones (Scheme 9). Two compounds, bearing an *N*-ethyl and either a 4′-fluorophenyl (**47**) or a 4′-chlorophenyl (**48**) as the A ring of the chalcone showed the best topo II inhibition at 20 µM. Mechanism investigations demonstrated that these hybrids may function as non-intercalative topo II catalytic inhibitors. However, this study highlighted no correlation between the topo II inhibitory activity and cytotoxic effect. Therefore, further assays concerning the cell cycle distribution and annexin V binding were performed to study their action on cancer cells and evidenced apoptosis induction.

**Scheme 10:**
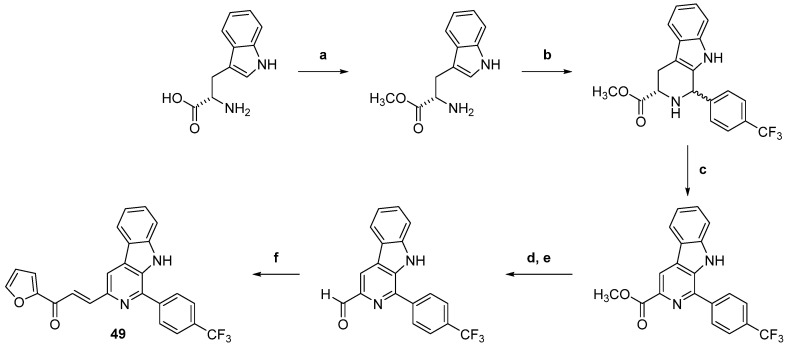
Synthesis of compound **49**. Reagents: (**a**) SOCl_2_, MeOH; (**b**) 4-(trifluoromethyl)benzaldehyde, *p*-TsOH, toluene; (**c**) sulfur, xylene; (**d**) LiAlH_4_, THF; (**e**) Dess–Martin periodinane, DCM; (**f**) 2-acetylfuran, Ba(OH)_2_, MeOH. Adapted from Ref. [73].

Another heterocycle has been widely explored as an anti-topoisomerase agent: carbolines, which present an additional nitrogen atom compared to carbazoles, are based on an indole ring fused with a pyridine one. β-carboline represents the basic scaffold for numerous alkaloids and many synthetic compounds designed for anticancer effects via multiple mechanisms, especially topoisomerase inhibition [72]. Thus, Kamal et al. designed a series of chalcone-linked β-carboline hybrids to be original topo I inhibitors [73]; they considered previous structure–activity relationship analysis revealing that the introduction of appropriate substituents at the C1 and C3 positions of the β-carboline ring increased antitumor activity as well as DNA binding ability [74]. Therefore, they synthesized, via a multistep pathway, some hybrids by placing a substituted phenyl at the C1 position and a chalcone part at the C3 position. Initially, a Pictet–Spengler condensation of L-tryptophan methyl ester with various substituted benzaldehydes yielded the corresponding 1,2,3,4-tetrahydro β-carboline derivatives. Their aromatization was achieved using sulfur in xylene to form the corresponding methyl β-carboline-3-carboxylate derivatives. The ester group at the C3 position of these intermediates was then reduced to its corresponding alcohol by lithium aluminum hydride in dry THF. These primary alcohols were further oxidized using Dess–Martin periodinane to afford 1-phenyl-3-carboxaldehyde β-carboline derivatives. Finally, the latter was reacted with a variety of aromatic as well as heteroaromatic ketones through a barium hydroxide-mediated Claisen–Schmidt condensation to give chalcone-linked β-carboline hybrids (Scheme 10). Among all of the hybrids synthesized, one derivative (**49**) bearing a 4-trifluoromethyl substituent at the C1-phenyl and a furan-containing chalcone moiety at C3 displayed a potent cytotoxic effect against several cancer cell lines, correlated to a moderate inhibition of topo I. To gain further insight into the binding mode of these hybrids to topo I, molecular docking studies were performed. In particular, the nitrogen atom in the last β-carboline ring showed a hydrogen bonding interaction with the guanidine group of Arg364 while the furan ring was stacked with Cys112, with the enone region forming interactions with Ala113.

**Scheme 11:**
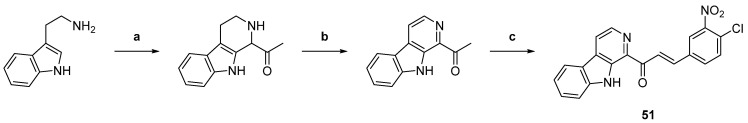
Synthesis of compound **51**. Reagents: (**a**) pyruvic aldehyde, 1% H_2_SO_4_; (**b**) KMnO_4_, DMF; (**c**) 4-chloro-3-nitro-benzaldehyde, NaOH, anhydrous EtOH. Adapted from Ref. [76].

The β-carboline scaffold is found in many alkaloids such as harmine (**50**), which occurs in a number of different plants including Syrian rue (*Peganum harmala* L.) and presents a variety of biological properties, especially anticancer effects [75]. Very recently, Guo et al. reported a series of harmine–chalcone hybrids: the 1-methyl substituent of harmine was replaced by a chalcone moiety; this hybridization was based on the merging strategy, with the A ring of chalcone corresponding to the β-carboline nucleus. Three steps of synthesis were necessary starting from tryptamine or 5-methoxytryptamine; first, these two compounds reacted with acetaldehyde in acidic medium and the resulting intermediates, bearing a methylketone moiety, were subsequently oxidized with potassium permanganate to give the corresponding β-carbolines. The methylketone part allowed the aldol condensation with different benzaldehydes in alkaline medium to afford the desired harmine–chalcone hybrids (Scheme 11). Guo et al. evaluated their antiproliferative activities against six human cancer cell lines: MCF-7, MDA-MB-231 (breast), HepG2, HT29, A549 and PC-3 [76]. The most potent derivative (**51**) was further studied and its ability to inhibit topo I was demonstrated (dose-dependent inhibition 25 to 100 µM). Additional assays proved that this compound was acting as a topo I poison like CPT, and that its inhibitory effect was superior to that of CPT. A molecular docking study revealed that its molecular structure overlapped with that of CPT and hydrogen bonding interactions were identified, especially between Cl and NO_2_ substituents with amino acid residues THR718 and DG12, respectively.

**Scheme 12:**
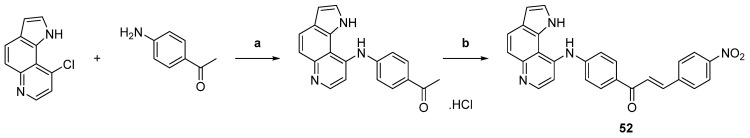
Synthesis of compound **52**. Reagents: (**a**) HCl, MeOH; (**b**) 4-nitro-benzaldehyde, 50% NaOH, MeOH. Adapted from Ref. [77].

Then, Via et al. developed three hybrids by combining a chalcone core with a pyrroloquinoline unit through an amino linker on the 4′-position of the chalcone A ring [77]. The design of such hybrids was based on a previous study dealing with DNA-targeted alkylating pyrroloquinolines [78]. 9-chloro-pyrrolo[3,2-f]quinoline reacted with *p*-amino-acetophenone in the presence of HCl as a catalyst, according to a nucleophilic substitution, to afford an acetyl-aniline derivative as a mono-hydrochloride. The latter led to the expected compounds when submitted to Claisen–Schmidt condensation with the appropriate aromatic aldehydes. The B rings of the chalcone hybrids were 2-pyrrolyl, 2-thienyl and 4-nitrophenyl (Scheme 12). The capacity of the pyrroloquinoline derivatives to intercalate between base pairs was linked to the inhibition of the supercoiled DNA relaxation by topo II, compound **52** (with a 4-nitrophenyl) being the most potent of the series (inhibition at 10 µM). However, these compounds induced no significant DNA cleavage. It should be noted that the corresponding chalcones without a pyrroloquinoline unit were inactive against topo II.

**Scheme 13:**
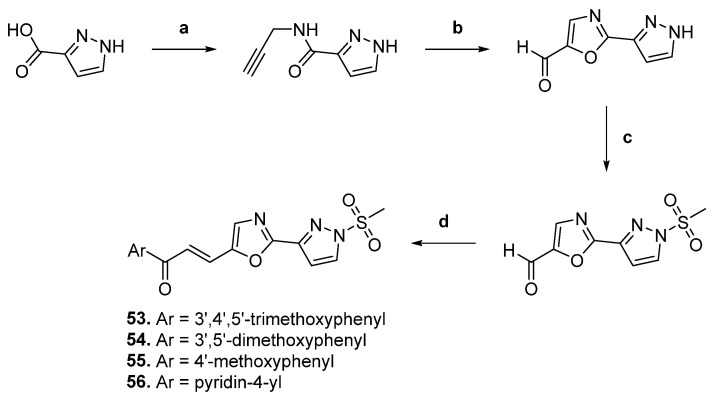
Synthesis of compounds **53** to **56**. Reagents: (**a**) propargylamine, DIPEA, HATU, THF; (**b**) mercuric perchlorate, ceric ammonium nitrate, acetonitrile; (**c**) methanesulfonyl chloride, DIPEA, DCM; (**d**) appropriate acetophenones, piperidine, EtOH. Adapted from Ref. [79].

Finally, Rangaswamy et al. were interested in 10 oxazole-linked pyrazole chalcones (Scheme 13) [79]. A review has covered the literature about studies dealing with the design of highly efficient oxazole-based anticancer drugs [80]; some of these compounds were found to target DNA topoisomerases. Reaction between 1*H*-pyrazole-3-carboxylic acid and prop-2-yn-1-amine in the presence of HATU and DIPEA led to *N-*(prop-2-yn-1-yl)-1*H*-pyrazole-3-carboxamide This key intermediate was reacted with Hg(ClO_4_)_2_ and CAN in acetonitrile at room temperature to afford 2-(1*H*-pyrazol-3-yl)oxazole-5-carbaldehyde. Further, reaction of this compound with methane sulfonyl chloride in the presence of DIPEA furnished the *N-*methylsulfonyl-pyrazolyl derivative. Finally, this intermediate underwent condensation reactions with differently substituted aromatic ketones by using piperidine as a catalyst at reflux conditions (ethanol was used as the solvent) to give the final oxazole-linked pyrazole chalcone derivatives. Among these compounds, six were active against SiHa (squamous cell carcinoma of the uterine cervix), A549, MCF-7, and Colo-205 (colorectal adenocarcinoma) cancer cell lines (IC_50_ ranging from 0.01 to 2.54 µM). Docking using human topo II as the biological target demonstrated that four molecules (**53–56**) were highly able to develop strong interactions with this enzyme. The interaction outlines of these compounds with the active site residues of human topoisomerase IIα protein were similar to those of etoposide; thus, the latter and compound **56** with a pyridine ring exhibited a close alignment, particularly between the carbonyl group of etoposide and the sulfonyl group of compound **56**. This one was able to mimic the critical hydrogen bond interactions observed for etoposide with ASP479 and SER480 residues. Moreover, for the chalcone hybrid **56**, the carbonyl of the enone moiety was found to form another hydrogen bond interaction with GLY776.

**Table 4:** Chalcone hybrids and reference substances as topoisomerases inhibitors.

C. No (Name)	Structure	Activity on Topo: IC_50_ (µM) or % Inhibition	Activity on Cancer Cells IC_50_ (µM)	Ref.
**36** (ciprofloxacin CP)	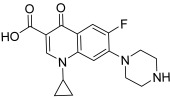	Topo II: 104 µM	Hela: 300 MG63: 480 K562: >150 NCI-H460: 60.0	[59]
**37**	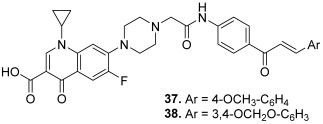	Topo I: 68% at 100 µM 25% at 20 µM Topo II: 85% at 100 µM 50% at 20 µM	-	[15]
**38**		Topo I: 91% at 100 µM 26% at 20 µM Topo II: 86% at 100 µM 44% at 20 µM	-	
**39**	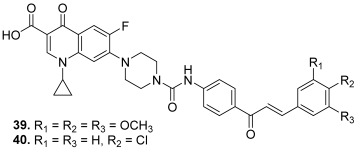	Topo I: 16.0 µM Topo IIA: 137 µM	HCT116: 2.0 SR: 0.6	[16]
**40**		Topo I: 18.0 µM Topo IIB: 107 µM	HCT116: 2.5 SR: 0.7	
		Topo I: 37.5 µM Topo II: 19.9 µM	HCT116: 5.0 LOX IMVI: 1.3	[60]
**41**	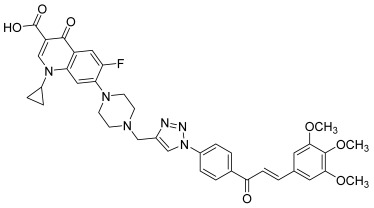	Topo I: 25% at 2.5 µM Topo IIB: 100% at 100 µM 95% at 10 µM	Caco-2: 7.1 HCT116: 2.5 HT-29: 13.2 RPMI-8226: 0.4 µM	[61]
**42**	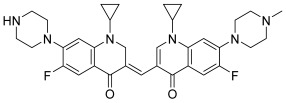	Topo II: Partial inhibition at 1.6 μM, with Virtually complete inhibition at 3.2 μM	BGC-823: 3.8 Capan-1: 2.9 HGC-27: 5.6 Panc-1: 3.5 DU145: 3.2 T24: 3.7	[63]
**43**	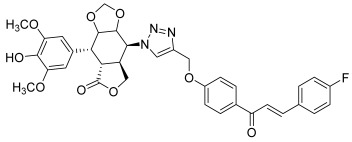	Topo II	Colo-205: 12.5 DU145: 12.0 HCT-15: 12.4 HeLa: 4.5 SKN-SH: 0.4 SW-620: 0.4	[65]
**44**	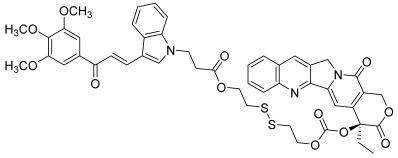	Topo I:	A549: 0.4 A549/PTX: 0.6 HCT116: 0.2 HCT116/PTX: 0.3	[66]
		Without GHS: moderate activity at 20 µM With GSH (1 mM): moderate activity at 20 µM/high activity at 40 µM		
**45**	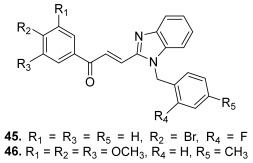	Topo II: 87% at 20 µM	A549: 3.6 HePG2: 4.5 MG63: 4.7 LNCaP: 5.4	[68]
**46**		Topo II: 95% at 20 µM	A549: 3.8 HePG2: 4.6 MG63: 4.1 LNCaP: 3.6	
**47**	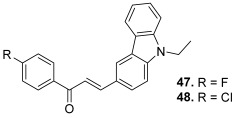	Topo II: inhibition at 20 µM.	A549: 16.7 HeLa: >50 HL-60: 12.9 PC-3: 32.3	[71]
**48**		Topo II: inhibition at 20 µM.	A549: 9.6 HeLa: 5.5 HL-60: 2.9 PC-3: 7.1	
**49**	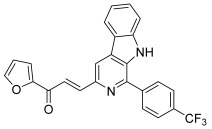	Topo I: inhibition at 100 µM	A549: 2.6 ACHN: 2.13 DU145: 3.3 MCF-7: 1.9 HeLa: 2.8	[73]
**50** (harmine)	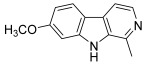	Topo I: inhibition at 150 µM	-	[73,75]
**51**	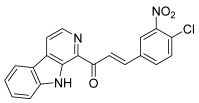	Topo I: dose-dependent inhibition 25 to 100 µM	A549: 2.0 HepG2: 1.6 HT29: 0.6 MCF-7: 0.3 MDA-MB-231: 1.0 PC-3: 1.2	[76]
**52**	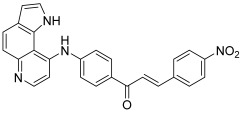	Topo II: inhibition at 10 µM.	HeLa: 10.4 HL-60: 4.4 JR8: 1.2	[77]
**53**	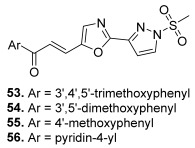	Topo II	A549: 0.1 Colo-205: 0.1 MCF-7: 0.1 SiHa: 0.2	[79]
**54**			A549: 0.9 Colo-205: 1.4 MCF-7: 1.2 SiHa: 0.9	
**55**			A549: 1.7 Colo-205: 2.0 MCF-7: 1.8 SiHa: 1.7	
**56**			A549: 0.01 Colo-205: 0.04 MCF-7: 0.07 SiHa: 0.1	

## 4. Conclusions

Topoisomerases are essential enzymes involved in key cellular processes such as DNA replication and transcription. Therefore, these enzymes have emerged as promising anticancer targets and many inhibitors have been developed. Some of them are considered to be major anticancer agents, such as anthracyclines, etoposide or irinotecan, which are nature-inspired compounds.

Chalcones are polyphenolic compounds belonging to the vast family of flavonoids, widely distributed in the plant kingdom. They are open-chain molecules in which two aromatic rings are joined by a three-carbon α,β-unsaturated carbonyl. Chalcones are well-known to exert a variety of biological activities. Thus, many compounds with a chalcone structure exhibit great anticancer effects through interference with different mechanisms and targets such as aromatase, VEGF, tubulin and topoisomerases.

This review pointed out some natural chalcones as potent topo inhibitors but their number remains quite limited. Most of these chalcones have hydroxyl or methoxy substituents on their aromatic rings, which were sometimes found to form hydrogen bonds with amino acid residues of the topo active site. Π–π interactions with DNA residues were also highlighted, perhaps owing to the electron-donating effect from these oxygenated groups.

Considering synthetic chalcones, here again hydroxyl or methoxy substituents were proven to be of interest; the presence of an additional chlorine atom seemed to be favorable to interactions with topoisomerase I or II. The replacement of the benzene ring by a heterocyclic structure was also found to enable inhibitory effects toward topoisomerases. Nevertheless, in a series, a thiophene core instead of a furan one led to totally inactive compounds. Several examples of furan derivatives demonstrated their anti-topo activity, including the chalcone hybrid **49** bearing a carboline unit. Besides, the introduction of small heterocycles such as epoxide or thioepoxide or even pyrrolidine on aromatic rings was shown to increase their inhibitory potency against topoisomerases; in fact, epoxide-substituted chalcones were found to selectively inhibit topo I while thioepoxide-substituted chalcones were shown to be selective topo II inhibitors. These findings highlight the structural diversity of chalcones as topoisomerases inhibitors but also the difficulty of establishing structure–activity relationship elements. Finally, this review underlines recent advances in hybridization strategies to develop promising topoisomerase inhibitors and above all multi-target drugs. In this context, ciprofloxacine–chalcone hybrids are of great interest due to their inhibitory effect against both topo I and II, while ciprofloxacine is only a topo II inhibitor. In these hybrids, the linker’s nature seemed to influence the inhibitory effect, with the triazole pattern leading to the most active compounds. Nitrogen heterocycles-based hybrids also appeared to be potent topoisomerases inhibitors; it is worth noticing that carbazole derivatives were topo II inhibitors while chalcone–carboline hybrids (with an additional nitrogen compared to carbazole) were active against topo I. Other hybrids demonstrated notable potential as, in addition to their anti-topo effect, they acted on biological targets involved in entirely separate stages of the cell division process, such as inhibition of tubulin polymerization by compound **44**.

In brief, even if structure–activity relationship elements are not evident, some structural features seemed to be important for topoisomerases inhibition. First, some studies pointed out the role of the carbonyl group of chalcone in the hydrogen bond formation with amino acid residues. Besides, it is also worth considering the substituents of aromatic rings, which can influence the ability of chalcones to establish hydrogen bonds with the active site of topoisomerases or to reinforce π–π interactions between these aromatic rings and DNA residues. In addition, the heterocyclic nature of some inhibitors appeared to be essential for their anti-topo effect. Finally, further studies on both types of these enzymes appear necessary to better understand which structural modifications of the chalcone core could enhance selectivity toward one or another of the topoisomerases.

To conclude, the findings presented herein support the continued exploration of chalcones as promising scaffolds in the design of novel anticancer therapeutics, especially via topoisomerase inhibition.

## Data Availability

No new data were created or analyzed in this study.

## Abbreviations

The following abbreviations are used in this manuscript:

CPT	Camptothecin
CuAAC	Copper-Catalyzed Azide-Alkyne Cycloaddition
CP	Ciprofloxacin
DC	Deoxycytidine
DCM	Dichloromethane
DG	Deoxyguanosine
DIPEA	*N,N*-diisopropylethylamine
DMAP	4-ddimethylaminopyridine
DMF	Dimethylformamide
DNA	Deoxyribonucleic acid
DOX	Doxorubicin
EDCI	1-ethyl-3-(3-dimethylaminopropyl)carbodiimide
GSH	Glutathione
HATU	Hexafluorophosphate azabenzotriazole tetramethyl uronium
HOBt	Hydroxybenzotriazole
IARC	International Agency for Research on Cancer
IC_50_	Half maximal inhibitory concentration
ISL	Isoliquiritigenin
MDAs	Microtubule destabilizing agents
MDR	Multidrug resistance
MTDs	Multi-targeted drugs
topo	Topoisomerase
**Cancer Cell Lines**	
The following cancer cell lines are used in this manuscript:	
A2780	Ovarian endometrioid adenocarcinoma
A2780CP	Cisplatin-resistant A2780
A2780S	Cisplatin-sensitive A2780
A549	Lung adenocarcinoma
A549/PTX	Paclitaxel-resistant A549
ACHN	Papillary renal cell carcinoma
AZ521	Gastric carcinoma
BGC-823	Human papillomavirus-related endocervical adenocarcinoma
BT474	Invasive breast carcinoma of no special type
Caco-2	Colon adenocarcinoma
Capan-1	Pancreatic ductal adenocarcinoma
Colo-205	Colon adenocarcinoma
CRL-1579	Amelanotic melanoma
DH82	Canine histiocytic sarcoma
DLD-1	Colon adenocarcinoma
DU145	Prostate carcinoma
HCT116	Colon carcinoma
HCT116/PTX	Paclitaxel-resistant HCT116
HCT-15	Colon adenocarcinoma
Hep-2	Human papillomavirus-related endocervical adenocarcinoma
HepG2	Hepatoblastoma
HGC-27	Hepatoblastoma
HL-60	Adult acute myeloid leukemia
HT-1376	Bladder carcinoma
HT-29	Colon adenocarcinoma
HuCCA-1	Cholangiocarcinoma
JR8	Melanoma
Jurkat	Childhood T acute lymphoblastic leukemia
K562	Chronic myeloid leukemia
LNCaP	Prostate carcinoma
LOX-IMVI	Amelanotic melanoma
LS174T	Colon adenocarcinoma
MCF-7	Invasive breast carcinoma of no special type
MDA-MB-231	Breast adenocarcinoma
MDA-MB-468	Breast adenocarcinoma
MG63	Osteosarcoma
MOLT-3	Adult T acute lymphoblastic leukemia
NCI-H460	Lung large cell carcinoma
NCI-H460/R	Doxorubicin-resistance NCI-H460
Panc-1	Pancreatic ductal adenocarcinoma
PC-3	Prostate carcinoma
RPMI-8226	Multiple myeloma
SiHa	Human papillomavirus-related cervical squamous cell carcinoma
SK-MEL-2	Melanoma
SKN-SH	Neuroblastoma
SK-OV-3	Ovarian serous cystadenocarcinoma
SR	Anaplastic large cell lymphoma
SNU475	Adult hepatocellular carcinoma
SNU638	Gastric adenocarcinoma
SW-620	Colon adenocarcinoma
T24	Bladder carcinoma
T47D	Invasive breast carcinoma of no special type
U87	Glioblastoma
